# Computed tomography-based radiomics nomogram for prediction of lympho-vascular and perineural invasion in esophageal squamous cell cancer patients: a retrospective cohort study

**DOI:** 10.1186/s40644-024-00781-w

**Published:** 2024-10-04

**Authors:** Bin Tang, Fan Wu, Lin Peng, Xuefeng Leng, Yongtao Han, Qifeng Wang, Junxiang Wu, Lucia Clara Orlandini

**Affiliations:** 1 Department of Radiation Oncology, Radiation Onocology Key Laboratory of Sichuan Province, Sichuan Cancer Hospital & Institute, Affiliated Cancer Hospital of University of Electronic Science and Technology of China, Chengdu, 610041 China; 2 Department of Thoracic Surgery, Radiation Oncology Key Laboratory of Sichuan Province, Sichuan Cancer Hospital & Institute, Affiliated Cancer Hospital of University of Electronic Science and Technology of China, Chengdu, 610041 China

**Keywords:** Esophageal squamous cell cancer, Lympho-vascular invasion, Perineural invasion, Contrast-enhanced CT, Radiomic, LASSO

## Abstract

**Purpose:**

Lympho-vascular invasion (LVI) and perineural invasion (PNI) have been established as prognostic factors in various types of cancers. The preoperative prediction of LVI and PNI has the potential to guide personalized medicine strategies for patients with esophageal squamous cell cancer (ESCC). This study investigates whether radiomics features derived from preoperative contrast-enhanced CT could predict LVI and PNI in ESCC patients.

**Methods and materials:**

A retrospective cohort of 544 ESCC patients who underwent esophagectomy were included in this study. Preoperative contrast-enhanced CT images, pathological results of PNI and LVI, and clinical characteristics were collected. For each patient, the gross tumor volume (GTV-T) and lymph nodes volume (GTV-N) were delineated and four categories of radiomics features (first-order, shape, textural and wavelet) were extracted from GTV-T and GTV-N. The Mann–Whitney U test was used to select significant features associated with LVI and PNI in turn. Subsequently, radiomics signatures for LVI and PNI were constructed using LASSO regression with ten-fold cross-validation. Significant clinical characteristics were combined with radiomics signature to develop two nomogram models for predicting LVI and PNI, respectively. The area under the curve (AUC) and calibration curve were used to evaluate the predictive performance of the models.

**Results:**

The radiomics signature for LVI prediction consisted of 28 features, while the PNI radiomics signature comprised 14 features. The AUCs of the LVI radiomics signature were 0.77 and 0.74 in the training and validation groups, respectively, while the AUCs of the PNI radiomics signature were 0.69 and 0.68 in the training and validation groups. The nomograms incorporating radiomics signatures and significant clinical characteristics such as age, gender, thrombin time and D-Dimer showed improved predictive performance for both LVI (AUC: 0.82 and 0.80 in the training and validation group) and PNI (AUC: 0.75 and 0.72 in the training and validation groups) compared to the radiomics signature alone.

**Conclusion:**

The radiomics features extracted from preoperative contrast-enhanced CT of gross tumor and lymph nodes have demonstrated their potential in predicting LVI and PNI in ESCC patients. Furthermore, the incorporation of clinical characteristics has shown additional value, resulting in improved predictive performance.

**Supplementary Information:**

The online version contains supplementary material available at 10.1186/s40644-024-00781-w.

## Introduction

Lympho-vascular invasion (LVI) and perineural invasion (PNI) have been established as prognostic factors in various types of cancers, such as breast cancer [1], colorectal cancer [2], lung cancer [3, 4], gastric cancer [5, 6] and also in esophageal cancer (EC) [7–10]. It is noteworthy that approximately 90% of EC were diagnosed with esophageal squamous cell cancer (ESCC) [11], and the incidence of PNI and LVI in ESCC patients was around 20% or even higher [10, 12]. Moreover, the presence of LVI and/or PNI is often associated with an increased risk of micrometastasis and recurrence or poor overall survival (OS) and progression-free survival (PFS) [13–17]. Although the pathological description and clinical significance are clearly understood, the exploration of the underlying molecular mechanism is ongoing [18].

The diagnosis of LVI and PNI currently relies on postoperative histopathology, and typically the administration of postoperative adjuvant therapy can be considered based on the histopathological findings obtained from surgery [19]. It’s worth noting that the treatment of ESCC is still a multidisciplinary challenge and preoperative prediction of LVI and PNI status is necessary for patients to implement an aggressive treatment plan [20]. Patients with suspected LVI or PNI require more comprehensive treatment, such as more extensive surgery or preoperative adjuvant therapy [21, 22]. The poor prognosis and rising incidence of esophageal cancer [23] highlight the need to improve pre-treatment diagnosis and prediction methods, as early identification of LVI and PNI is potentially useful in developing the most appropriate management pathways for ESCC patients. However, the preoperative identification of LVI and PNI remains difficult.

Radiomics, as an emerging field of data mining, involves the extraction of high-dimensional quantitative features from medical imaging. These extracted data are subsequently utilized for prediction, diagnosis, prognosis and longitudinal monitoring. Radiomic has gained popularity as a research trend and has demonstrated promising achievements in various types of diseases. Specifically, in the case of ESCC, radiomic studies have been conducted to predict treatment response [24, 25], prognosis [26] and classification of tumor. To the best of our knowledge, only a few studies have reported on the feasibility of using a radiomics-based approach for predicting LVI or PNI in ESCC [27–29], and the prediction both LVI and PNI is rare.

In this retrospective study, we first demonstrated the validity of radiomics signatures constructed from preoperative contrast-enhanced CT as reliable predictors for LVI and PNI in ESCC patients. Moreover, to achieve more accurate prediction, two nomogram models incorporating radiomics signatures with clinical characteristics were developed and validated. Based on the prediction model, the early identification of LVI and PNI can enable the stratification of ESCC patients thus potentially supports personalized clinical strategy in the era of precise medicine.

## Materials and methods

### Patients included and clinical workflow

**Fig. 1:**
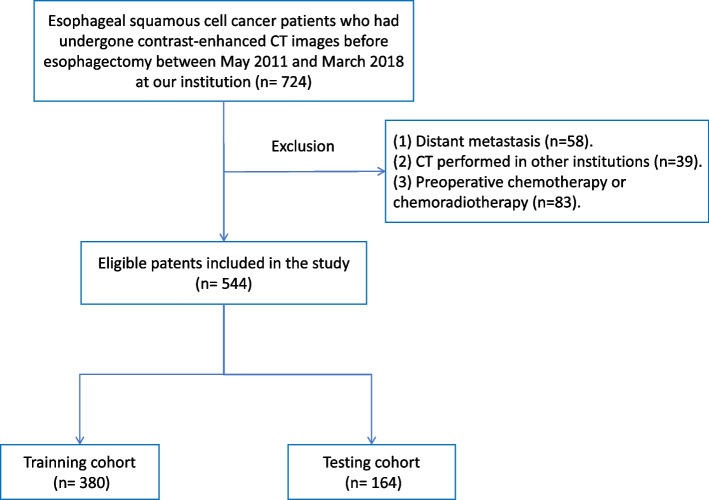
The flow chart of subject enrollment

A retrospective cohort of 544 patients with ESCC treated at our institution (Sichuan Cancer Hospital, China) between May 2011 and March 2018 were included in this study. All patients included underwent either surgery alone or surgery followed by adjuvant chemotherapy or concurrent chemoradiotherapy; the following inclusion criteria were adopted: (1) age above 18 years; (2) underwent standard McKeown esophagectomy or Ivor Lewis esophagectomy; (3) had preoperative contrast-enhanced CT imaging available; (4) had histopathological results for perineural and lympho-vascular invasion. Patients who had distant metastases, or CT performed in other institutions, or received preoperative chemotherapy/chemoradiotherapy were excluded, the flow chart of subject enrollment is depicted in Fig. 1. Patients were randomly divided into the training group and validation group at a ratio of 7 to 3. This study was approved by the ethics committee of Sichuan Cancer Hospital & Institution (Approval number: SCCHEC-02–2020-015).

Patient clinical information was obtained by conducting a comprehensive review of both clinical and pathological records. This information included various factors such as age, gender, blood routine examination parameters (including white blood cell, neutrophil**,** basophil, neutrophil to lymphocyte ratio [NLR] and high-sensitivity C-reactive protein [hs-CRP] et al.), as well as coagulation test results (including prothrombin time, thrombin time, activated partial thromboplastin time [APTT], international normalized ratio [INR], D-Dimer, fibrinogen and fibrinogen degradation product [FDP] et al.). The pathological Tumor Node Metastasis (TNM) stage was determined according to the American Joint Committee on Cancer (AJCC)/International Union Against Cancer (UICC) 8th edition of the Cancer Staging Manual.

Adjuvant treatment was administered to patients who exhibited conventional high-risk factors, such as T3/T4 stage, lymph node-positive status, perineural invasion, lympho-vascular invasion, R1/R2 resection, or poor tumor grade unless contraindicated due to other reasons. These patients underwent platinum-based standard chemotherapy 4–6 weeks after surgery. Intensity-modulated radiation therapy (IMRT) was conducted over 5–6 weeks, delivering a total dose of 50–54 Gy/25–30 fractions (5 fractions per week).

### Histopathologic examination

All sections were thoroughly examined by experienced pathologists specializing in esophagology to determine histological parameters, including tumor type, grade, tumor invasion depths, presence of lymph node metastasis and their count, and assessment of resection margins (classified according to the World Health Organization classification). The PNI and LVI status of each patient was evaluated based on the pathological results of the surgical specimen typically by Hematoxylin and Eosin (H&E) staining. For the determination of LVI, the presence of tumour emboli within either the lymphatic or vascular channels were considered as true invasion. Speicifically, peritumoral vessels outside the tumor circumference were considered peritumoral without a clearance distance, and only peritumoral vessel invasion was considered as a true invasion, while intratumoral vessel infiltration was not considered. Evidence of true invasion included the apparent presence of an endothelial lining in the vessel and the extension of the endothelial lining onto the intravascular tumor plug. Tumor-associated thrombus formation in infiltrated vessels was also considered affirmative. For the determination of PNI, it was defined as tumor cells located in the perineural space, typically with circumferential or near-circumferential involvement, or intraneural extension of tumor cells.

### CT images and region of interest delineation

All patients underwent contrast-enhanced CT imaging one week before surgery by GE Lightspeed VCT (GE Healthcare, Little Chalfont, UK), or Philips Brilliance iCT (Philips Medical solution, Cleveland, OH) CT scanners. Images were acquired with a delay of 50 s after the intravenous injection of an iodinated contrast medium using the following parameters: tube voltage of 120 kV, tube current of 230 mA, slice thickness of 5 mm without slice increment, and in-slice pixel dimension of 0.88 mm (range, 0.78–0.98 mm).

The gross target volume of the primary tumor (GTV-T) and lymph nodes (GTV-N) were delineated as regions of interest (ROIs) and reviewed by two experienced esophageal radiologists using the MIM Maestro workstation (MIM Software Inc, Cleveland, OH), following the guidelines outlined in ICRU n.83 [18]. The GTV-N was created by consolidating all discernible lymph nodes with a major axis greater than 5 mm, spanning from the supraclavicular to the left gastric lymph node region.

### Image preprocessing and features extraction

The original images were first pre-processed using wavelet filters and then decomposed into 8 decompositions per level by applying all possible combinations of a High (H) or a Low (L) pass filter in each of the three dimensions, i.e. HHH, HHL, HLL, HLH, LHH, LHL, LLH and LLL. Subsequently, four categories of commonly used radiomics features were extracted from the ROIs of GTV-T and GTV-N, including shape features, first-order features, textural features and wavelet features (Supplementary Material E1). Among these features, shape features characterize the surface features of the ROI; First-order features describe the distribution of voxel intensities within the ROI, encompassing measures such as Entropy, Kurtosis, and Skewness; Textural features can capture the textural information of the ROI, including Gray Level Co-occurrence Matrix (GLCM), Gray Level Dependence Matrix (GLDM), Gray Level Run Length Matrix (GLRLM), Gray Level Size Zone Matrix (GLSZM), and Neighboring Gray Tone Difference Matrix (NGTDM) capture the textural information of the ROI; Wavelet features capture spatial variations and texture details at multiple scales which makes it suitable for characterizing complex tissue textures and identifying subtle abnormalities.

### Features selection and model building

Z-score normalization was applied to all radiomics features. To construct the prediction models for PNI and LVI, we designated PNI and LVI status as the target outcomes to be predicted in turn. To address the issue of the high dimensionality of radiomics features, we initially utilized the Mann–Whitney U test to identify potentially predictive features for the outcomes. Each feature was compared between the invasion-negative and invasion-positive patient groups to evaluate its relevance to the outcomes. Features with *p*-values below 0.05 were deemed significant, while those with *p*-values equal to or greater than 0.05 were considered insignificant.

To further reduce the dimensionality of the feature set, we employed the least absolute shrinkage and selection operator (LASSO). LASSO utilizes a regularization process that shrinks the coefficients associated with each variable towards zero, effectively selecting the most significant features while discarding the less important ones. This was achieved by increasing the regularization factor λ and applying the tenfold cross-validation to determine the optimal set of features. Ultimately, the features and their corresponding coefficients that yielded the lowest "Binomial Deviance" in LASSO were utilized to construct the radiomics signature (Rad-score) as follows:

1 $$\text{Rad}-\text{score}= \sum_{i}^{n}{C}_{i}{X}_{i}+b$$

where *n* is the number of features, *X*_*i*_ and* C*_*i*_ represent each feature and their corresponding coefficient, and *b* is the intercept. The Rad-score can serve as a prediction model solely based on image features to distinguish between outcome-positive and outcome-negative patients.

To investigate the added value of clinical characteristics in predicting LVI or PNI, we first employed the Mann–Whitney U test (for continuous variables) or Pearson’s *χ*^2^ test (for categorical variables) on all clinical characteristics. The aim was to identify significant predictors associated with LVI or PNI (*p* < 0.05). Subsequently, the Rad-score and the identified significant clinical characteristics were integrated to develop two separate multivariate logistic regression models for LVI and PNI using the training cohort. Additionally, two nomograms were created to visualize the prediction of each outcome.

### Statistical analysis

The preprocessing of images and features extraction was conducted using the PyRadiomics V3.0.1 Python package. The two steps of feature selection, namly Mann–Whitney U test and LASSO were perfromed performed in R environment (version: 3.6.3) [30]. To evaluate the performance of the radiomics signature and nomogram models, the receiver operating characteristic (ROC) curves were generated and the area under the curve (AUC) was calculated to assess their discrimination ability, then the 95% confidence interval (CI) of AUC was estimated using Bootstrap method. The calibration curves were plotted to determine the goodness of nomograms. The decision curve analysis were performed to evaluate the effective of prediction models. For the comparasion of patients characteristics between training and validation group, independent sample t test or Mann-Whiteny U test were used for continous variable, and chi-square test was used for categorical variables. A *p*-value < 0.05 was deemed statistically significant in all the analysis.

## Results

### Patients characteristics

A total of 544 ESCC patients were enrolled in this study. The mean age of the cohort is 62 (range 37.2 ~ 85.5). The number of patients with LVI-positive or PNI-positive is 110 (20.2%) and 126 (23.2%), respetively. The cohort is randomly divided into training group (*N* = 380) and validation group (*N* = 164), and the detail of gender, age, pathology T stage, pathology N stage, pTNM (8th edition), LVI and PNI were listed in Table 1. No significant difference were found between two groups for clinical characteristics (*p* > 0.05).

**Table 1:** Characteristics of patients in the training and validation cohorts

Variables	Training group (*N* = 380)	Validation group (*N* = 164)	*P* value
**Gender**			0.238
Female	69(18.2%)	23 (14.1%)	
Male	311(81.8%)	141 (85.9%)	
Age(years)*	62.2 (37.2–85.5)	61.6 (38.1–85.5)	0.825
**Pathology T stage**			0.937
T0	8(2.1%)	2(1.2%)	
T1	27(7.1%)	12(7.3%)	
T2	71(18.7%)	34(20.7%)	
T3	229(60.3%)	96(58.5%)	
T4	45(11.8%)	20(12.2%)	
**Pathology N stage**			0.303
N0	174(45.8%)	61(37.2%)	
N1	113(29.7%)	59(36%)	
N2	56(14.7%)	27(16.5%)	
N3	37(9.7)	17(10.4%)	
**pTNM (8th edition)**			0.427
I	43(11.3%)	14(8.5%)	
II	122(32.3%)	46(28.0%)	
III	168(44.2%)	78(47.6%)	
IV	47(12.4%)	26(15.9%)	
**Lympho-Vascular Invasion**			0.970
Yes	77 (20.3%)	33(20.1%)	
No	303 (79.7%)	131(79.9%)	
**Perineural Invasion**			0.660
Yes	90(23.7%)	36(22%)	
No	290(76.3%)	128(78%)	

^*^median and the range in the parentheses

### Features selection and radiomics signature building

For each ESCC patient, a total of 1726 features were extracted from the GTV-T and GTV-N. Specifically for each ROI, there were 17 shape features, 19 first order features, 75 textural features and 752 wavelet features. In the initial features selection step, the Mann–Whitney U test identified 216 and 32 significant features (*p* < 0.05) with regard to LVI and PNI status, respectively. Subsequently, LASSO regularization with tenfold cross-validation further reduces the dimensionality of these significant features. As a result, 29 features for LVI and 14 features for PNI were selected with non-zero coefficients in the radiomics signatures (Supplementary material E2). Detailed information about the radiomics signatures can be found in supplementary materials E3 and E4, along with the Eq. (1) used to construct them.

Among the selected features with non-zero coefficients, the majority belonged to the wavelet category and were utilized in constructing the radiomics signatures for LVI and PNI. Only 2 features were directly extracted from the raw images and used in the PNI radiomics signature. Within the LVI radiomics signature, 6 out of 29 features were extracted from the GTV-T, while the remaining 23 features were extracted from the GTV-N. In the case of the PNI radiomics signature, 8 out of 14 features were extracted from the GTV-T, while the remaining 6 features were extracted from the GTV-N.

### Construction of nomogram models

**Fig. 2:**
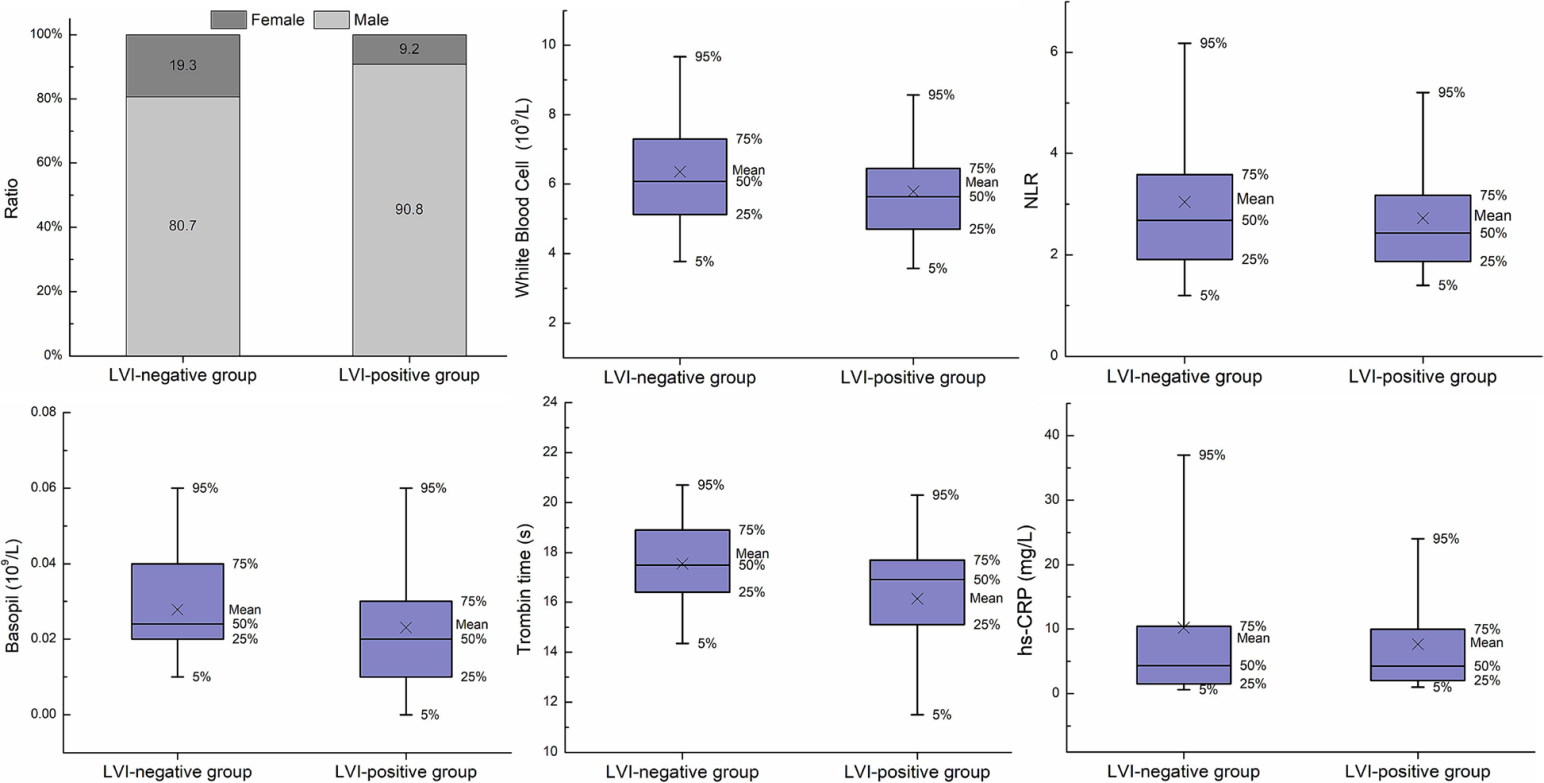
Comparison of selected clinical characteristics between LVI-negative and LVI-positive groups

**Fig. 3:**
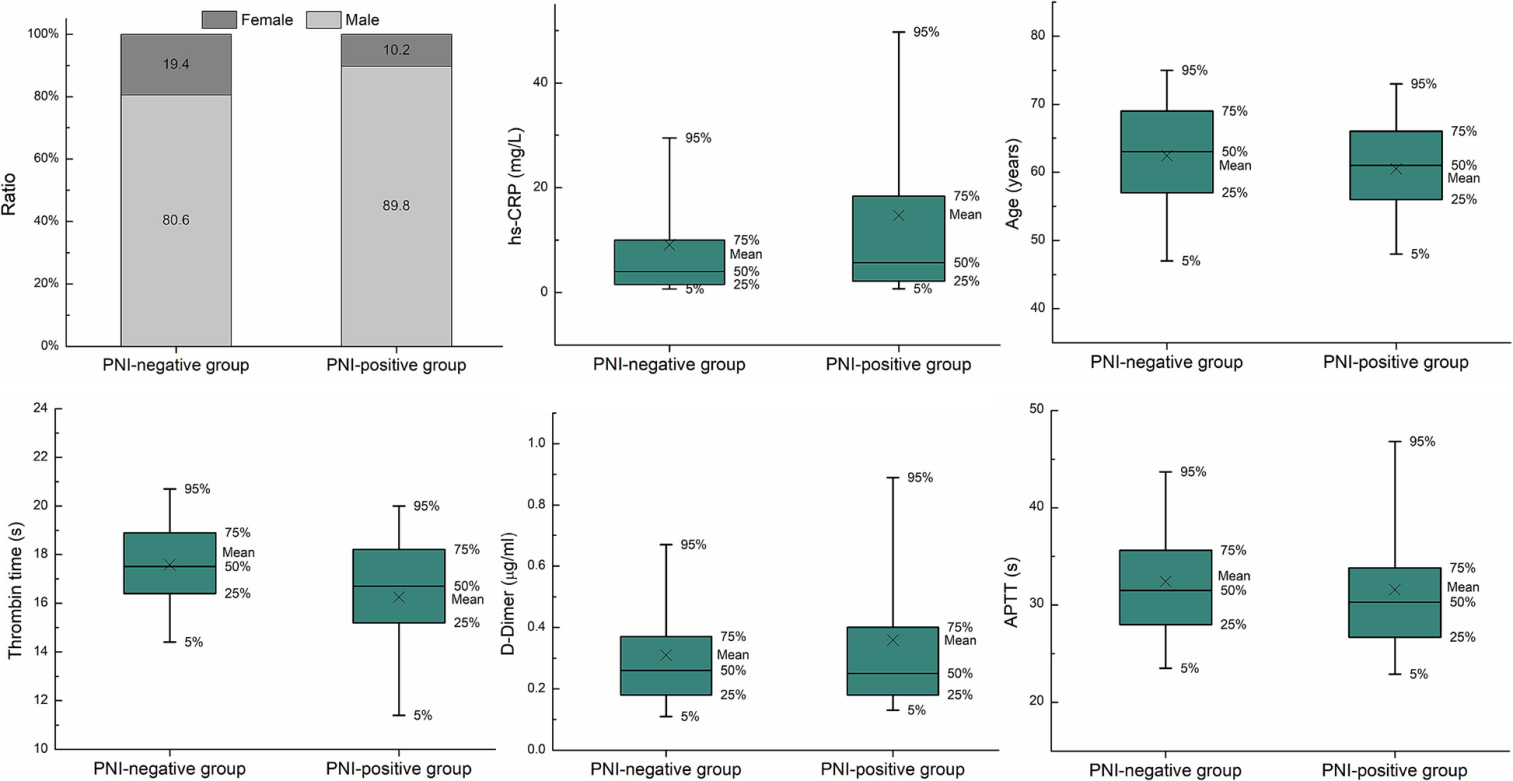
Comparison of selected clinical characteristics between PNI-negative and PNI-positive groups

**Fig. 4:**
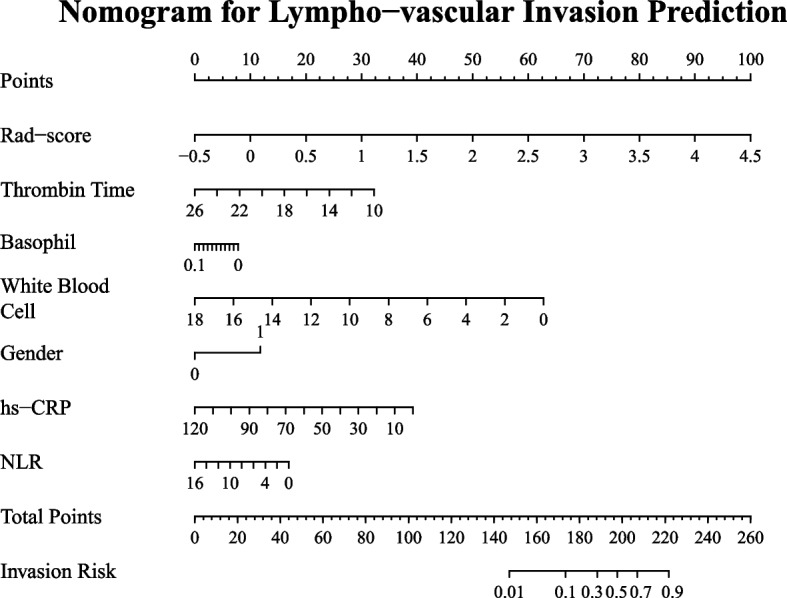
Nomogram for predicting LVI in ESCC. The nomogram was built in the training cohort with the Rad-score and clinical characteristics

**Fig. 5:**
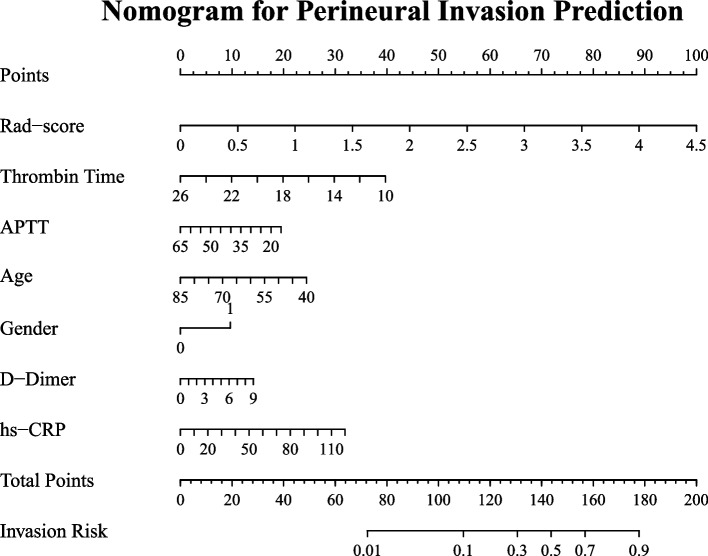
Nomogram for predicting PNI in ESCC. The nomogram was built in the training cohort with the Rad-score and clinical characteristics

Several clinical characteristics were found to be significant to either the patient's LVI status or PNI status, or both. Specifically, six clinical characteristics (thrombin time, gender, hs-CRP, basophil count, white blood cell count and NLR) exhibited a significant difference between the LVI-positive and LVI-negative groups. Similarly, six characteristics (thrombin time, gender, hs-CRP, age, D-Dimer and APTT) exhibited a significant difference between the PNI-positive and PNI-negative groups, as depicted in Figs. 2 and 3. Consequently, these significant clinical characteristics, along with the Rad-score, were utilized to construct a multivariate regression model known as the nomogram model, for the prediction of LVI and PNI, respectively. The nomograms were illustrated in Figs. 4 and 5.

### Predictive performance of radiomics signatures and nomogram models

**Fig. 6:**
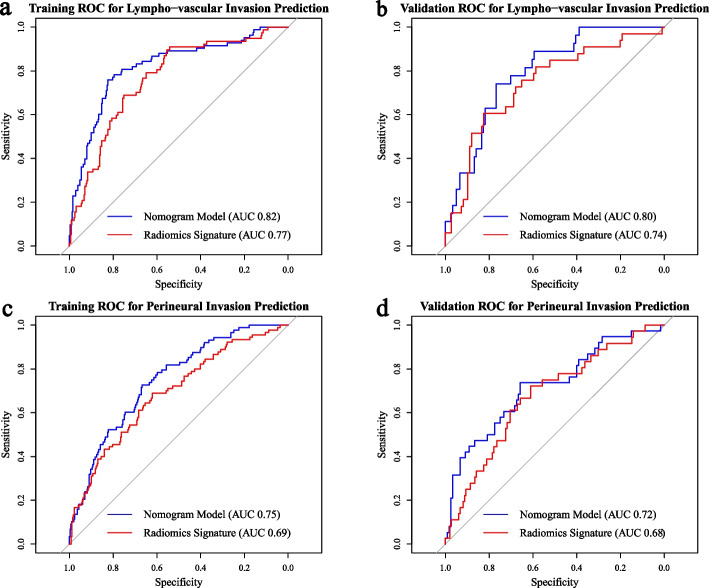
The ROC curves of lympho-vascular invasion (**a**, **b**) and perineural invasion prediction model (**c**, **d**) in the training and validation groups

**Fig. 7:**
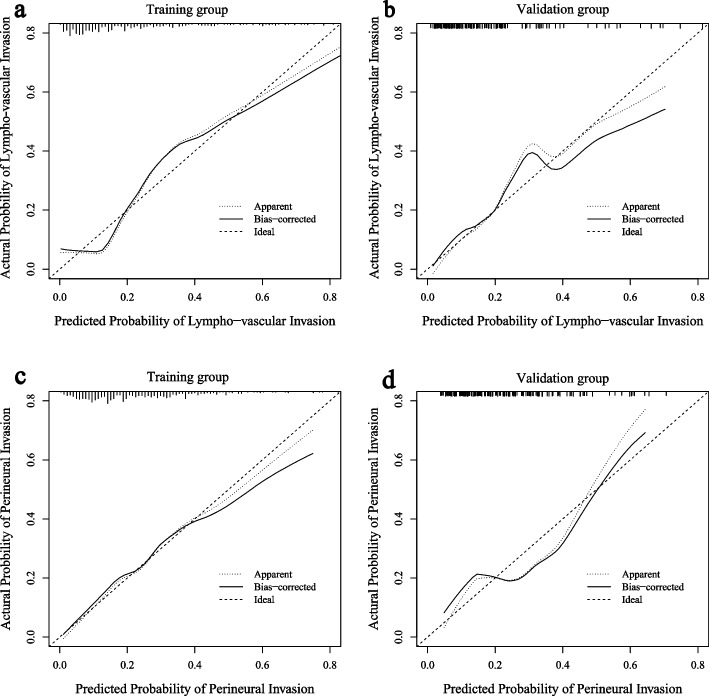
The calibration curves showing the nomogram-predicted vs actual prbability for lympho-vascular invasion (**a**, **b**) and perineural invasion prediction (**c**, **d**) in the training and validation groups

**Fig. 8:**
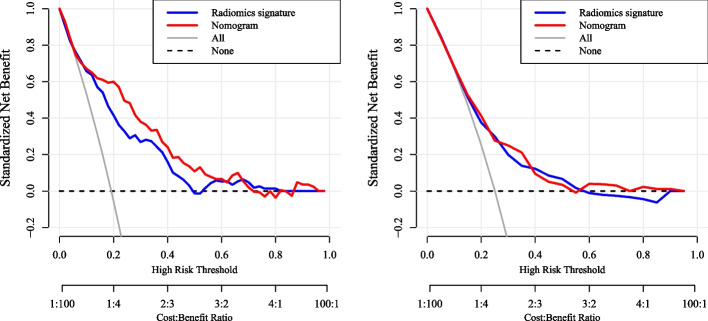
The decision curves for lympho-vascular invasion prediction (**a**) and perineural invasion prediction (**b**)

Both the radiomics signatures (Rad-score) and the nomogram models demonstrated a significant ability to distinguish between outcome-positive and outcome-negative patients (*p* < 0.05). Figure 6 illustrated the comparison of ROC curves between the radiomics signature and the nomogram models in both the training and validation groups, for LVI and PNI prediction, respectively. Incorporation of clinical characteristics resulted in improved predictive performance. The nomogram model achieved higher AUC values for LVI prediction in both the training group (AUC 0.82, 95% CI 0.77–0.87) and the validation group (AUC 0.80, 95% CI 0.71–0.88), outperforming the radiomics signature alone (training: AUC 0.77, 95% CI 0.71–0.82; validation: AUC 0.74, 95% CI 0.64–0.84). Similarly, for PNI prediction, the nomogram model exhibited better performance (training: AUC 0.75, 95% CI 0.69–0.80; validation: AUC 0.72, 95% CI 0.62–0.82) compared to the radiomics signature alone (training: AUC 0.69, 95% CI 0.63–0.75; validation: AUC 0.68, 95% CI 0.58–0.77). The detailed diagnostic performance of all the models can be found in Table 2. The calibration curves showing nomogram-predicted probability vs actual probability for LVI and PNI were plotted in Fig. 7. The x-axis represented the predicted LVI or PNI probability by nomogram and y-axis represented the actural probability. Perfect prediction would correspond to the “ideal” line, and the “apparent” dashed line represented the actual performance of the nomograms. The calibration curves indicated that the models make accurate predictions. Figure 8 showed the decision curve analysis of nomogram and radiomic signature for LVI and PNI prediction. Overall, the nomograms show better net benefits than radiomic signature for both predictions.

**Table 2:** Diagnostic performance of LVI and PNI prediction models in training and validation groups

Prediction Model/Evaluation Metric		TPR	FPR	TNR	FNR	Accuracy	F1-Score	AUC(95%CI)
LVI Radiomics Signature	Training group	0.89	0.46	0.54	0.11	0.61	0.47	0.77 (0.71 - 0.82)
	Validation group	0.60	0.19	0.81	0.40	0.77	0.56	0.74 (0.64 - 0.84)
LVI Nomogram	Training group	0.76	0.18	0.82	0.24	0.81	0.63	0.82 (0.77 - 0.87)
	Validation group	0.74	0.33	0.77	0.26	0.76	0.53	0.80 (0.71 - 0.88)
PNI Radiomics Signature	Training group	0.69	0.39	0.61	0.31	0.63	0.49	0.69 (0.63 - 0.75)
	Validation group	0.71	0.38	0.62	0.29	0.64	0.43	0.68 (0.58 - 0.77)
PNI Nomogram	Training group	0.73	0.33	0.67	0.27	0.68	0.51	0.75 (0.69 - 0.80)
	Validation group	0.74	0.34	0.66	0.26	0.68	0.52	0.72 (0.62 - 0.82)

*TPR* True Positive Rate, *FPR* False Positive Rate, *TNR* True Negative Rate, *FNR* False Negative Rate, *LVI* Lympho-vascular Invasion, *PNI* Perineural Invasion, *AUC* Area Under the Curve

## Discussion

In recent years, several studies have focused on investigating the prognostic value of lympho-vascular and perineural invasion in ESCC [14, 16, 31, 32]. These studies have consistently demonstrated the association of LVI and PNI with local recurrence and poor prognosis, suggesting that preoperative prediction of LVI and PNI could potentially influence treatment strategies. In our present study, we aimed to develop prediction models for LVI and PNI based on preoperative contrast-enhanced CT in ESCC patients. Our results indicate that a combination of image features from the primary tumor and lymph nodes can effectively predict LVI and PNI. Notably, when combined with clinical characteristics, the predictive performance of the model can be improved compared to using the radiomics signature alone.

To the best of our knowledge, the prediction of LVI and PNI for malignancies is still relatively uncommon. Previous studies have explored predictive models for LVI in endometrial cancer [33], breast invasive ductal carcinoma [34], gastric cancer [35] and rectal cancer [36], as well as for PNI in breast cancer [37, 38], colorectal cancer [39], gastric cancer [40]. In the case of ESCC, Li et al. incorporated clinical characteristics (cN stage and maximum tumor thickness) into the radiomics model based on contrast-enhanced CT to predict the LVI in 334 ESCC patients, achieving a AUC of 0.867 [27]. Zhou et al. developed a radiomics nomogram to predict the PNI of 360 ESCC patients, achieving an AUC of 0.803 [29]. The main differences between this study and previous studies are: 1) we simultaneously predicted LVI and PNI in ESCC patients while most of previous studies only predicted LVI or PNI for ESCC. Predicting both would be more beneficial for patients stratification; 2) the images features were extracted from the delineations of gross tumor volume and lymph-nodes, this fact might help to explore more potential predictive factors.

In this study, wavelet filters were used to process the contrast-enhanced CT images, which turned out a suitable choice as the selected radiomics features were predominantly extracted from the wavelet-filtered images. Wavelet transformation decomposes an image into wavelet coefficients, enabling the capture of information related to edges, textures, and other image characteristics. This approach provides a rich set of features that can enhance the discriminative power of radiomic-based models, potentially leading to improved disease characterization and prediction [41–43].

Clinical characteristics have demonstrated their ability to enhance prognostic prediction, detection, or classification in various tumor types [44–46]. Therefore, we also investigated the added value of clinical characteristics including gender, age, blood test result and coagulation function tests. Some of these variables were successfully combined with image features to construct the nomograms for LVI and PNI predictions. Gender and hs-CRP are common variables incorporated into both nomograms. CRP, a marker for inflammation, can interact with inflammatory and stromal cells in the tumor microenvironment, reflecting tumor cell proliferation, metastasis and overall cancer risk and prognosis [47, 48]. NLR, another inflammation marker, was included in the PNI nomogram prediction model. With regard to coagulation factors, thrombin time, APTT and D-Dimer were found to be significant and incorporated in the LVI nomogram prediction model, while thrombin time was also used for PNI prediction. Previous studies have shown an association between coagulation factors and disease progression [49, 50]. The underlying principle is that coagulation factors may interact with cancer cells and affect their development, growth and metastasis [51, 52].

There are several limitations in our study. Firstly, the cohort in this study was obtained from one single institution in China, thus no external validation was applied. Secondly, the resolution of contrast-enhanced CT in this study is relatively coarse. Therefore, conducting a prospective study with image quality as high as 3 mm or 2 mm slice thickness may potentially yield superior results.

## Conclusion

The radiomics features extracted from the gross tumor and lymph nodes on preoperative contrast-enhanced CT have demonstrated their potential in predicting LVI and PNI in ESCC patients. Furthermore, the incorporation of clinical characteristics has shown additional value, resulting in improved predictive performance.

## Supplementary Information

Supplementary Material 1.

## Data Availability

Research data are stored in an institutional repository and will be shared upon request to the corresponding author.
