# Supplementary material for: Computed tomography-based radiomics nomogram for prediction of lympho-vascular and perineural invasion in esophageal squamous cell cancer patients: a retrospective cohort study
Source: Cancer Imaging. 2024 Oct 4;24:131. doi: 10.1186/s40644-024-00781-w (PMC11451056; doi:10.1186/s40644-024-00781-w)
Supplement: Supplementary file 1 — Supplementary Material 1. [file 40644_2024_781_MOESM1_ESM.docx]

**Table E1.** Summaries of radiomics features extracted by Pyradiomic package

| Feature Class | Feature Name | Number |
| --- | --- | --- |
| Shape | Mesh Volume, Voxel Volume, Surface Area, Surface Area to Volume ratio, Compactness 1, Compactness 2, Spherical Disproportion, Maximum 3D diameter, Maximum 2D diameter (Slice), Maximum 2D diameter (Column), Maximum 2D diameter (Row), Major Axis Length, Minor Axis Length, Least Axis Length, Elongation, Flatness | 17 |
| First order | Energy, Total Energy, Entropy, Minimum, 10^th^ percentile, 90^th^ percentile, Maximum, Mean, Median, Interquartile Range, Range, Mean Absolute Deviation, Robust Mean Absolute Deviation, Root Mean Squared, Standard Deviation, Skewness, Kurtosis, Variance, Uniformity | 19 |
| Gray Level Co-occurrence Matrix (GLCM) | Autocorrelation, Joint Average, Cluster Prominence, Cluster Tendency, Contrast, Correlation, Difference Average, Difference Entropy, Difference Variance, Joint Energy, Joint Entropy, Informational Measure of Correlation 1, Informational Measure of Correlation 2, Maximal Correlation Coefficient, Inverse Difference Moment Normalized, Inverse Difference, Inverse Difference Normalized, Inverse Variance, Maximum Probability, Sum Average, Sum Entropy, Sum of Squares | 24 |
| Gray Level Size Zone Matrix (GLSZM) | Small Area Emphasis, Large Area Emphasis, Gray Level Non-Uniformity Normalized, Size-Zone Non-Uniformity, Size-Zone Non-Uniformity Normalized, Zone Percentage, Gray Level Variance, Zone Variance, Zone Entropy, Low Gray Level Zone Emphasis, High Gray Level Zone Emphasis, Small Area Low Gray Level Emphasis, Small Area High Gray Level Emphasis, Large Area Low Gray Level Emphasis, Large Area High Gray Level Emphasis, | 16 |
| Gray Level Run Length Matrix (GLRLM) | Short Run Emphasis, Long Run Emphasis, Gray Level Non-Uniformity, Gray Level Non-Uniformity Normalized, Run Length Non-Uniformity, Run Percentage, Gray Level Variance, Run Variance, Run Entropy, Low Gray Level Run Emphasis, High Gray Level Run Emphasis, Short Run Low Gray Level Emphasis, Short Run High Gray Level Emphasis, Long Run Low Gray Level Emphasis, Long Run High Gray Level Emphasis | 16 |
| Neighbouring Gray Tone Difference Matrix (NGTDM) | Coarse, Contrast, Busyness, Complexity, Strength | 5 |
| Gray Level Dependence Matrix (GLDM) | Small Dependence Emphasis, Large Dependence Emphasis, Gray Level Non-Uniformity, Dependence Non-Uniformity, Dependence Non-Uniformity Normalized, Gray Level Variance, Dependence Variance, Dependence Entropy, Low Gray Level Emphasis, High Gray Level Emphasis, Small Dependence Low Gray Level Emphasis, Small Dependence High Gray Level Emphasis, Large Dependence Low Gray Level Emphasis, Large Dependence High Gray Level Emphasis | 14 |
| Wavelet | Wavelet filtering yields 8 decompositions per level (all possible combinations of applying either a High or a Low pass filter in each of the three dimensions [x,y,z]), calculating first order statistics and textural features | 752 |

**Table E2. The number of significant and finally selected features for LVI and PNI radiomics signature**

| **Model** | **Number of significant features** | **Number of finally selected features with non-zero coefficients** |
| --- | --- | --- |
| LVI radiomics signature | 216 | 29 |
| PNI radiomics signature | 32 | 14 |

Note：LVI：Lympho-vascular Invasion; PNI: Perineural Invasion

**Table E3.** Significant features and their coefficients included in the radiomics signature for Lympho-vasular invasion prediction

| Feature Name | Feature Class | Wavelet Filter | ROI | Coefficient |
| --- | --- | --- | --- | --- |
| Gray Level Non-Uniformity Normalized | GLSZM | LHH | GTV-N | -0.2830 |
| Maximum Probability | GLCM | HHL | GTV-T | -0.2813 |
| Maximum | First order | HLL | GTV-N | 0.2671 |
| Long Run High Gray Level Emphasis | GLRLM | HHH | GTV-N | -0.2360 |
| Short Run High Gray Level Emphasis | GLRLM | HHL | GTV-N | -0.2151 |
| Interquartile Range | First order | LLL | GTV-N | -0.1936 |
| Dependence Variance | GLDM | LLL | GTV-N | 0.1751 |
| Complexity | NGTDM | HHL | GTV-T | -0.1693 |
| Coarseness | NGTDM | LHL | GTV-N | 0.1570 |
| Joint Energy | GLCM | LHL | GTV-T | -0.1513 |
| Large Dependence High Gray Level Emphasis | GLDM | LLH | GTV-N | -0.1398 |
| Inverse Variance | GLCM | HLL | GTV-N | 0.1168 |
| Dependence Non Uniformity Normalized | GLDM | HHH | GTV-N | -0.0922 |
| Correlation | GLCM | LLH | GTV-N | 0.0897 |
| Skewness | First order | LLH | GTV-N | -0.0886 |
| Zone Entropy | GLSZM | HLL | GTV-N | 0.0700 |
| Busyness | NGTDM | LLH | GTV-N | 0.0633 |
| Kurtosis | First order | HLH | GTV-N | 0.0588 |
| Dependence Variance | GLDM | HHL | GTV-T | 0.0583 |
| Maximum Probability | GLCM | HLH | GTV-N | -0.0430 |
| Large Dependence Low Gray Level Emphasis | GLDM | LLL | GTV-N | 0.0330 |
| Size Zone Non-Uniformity | GLSZM | HHH | GTV-T | 0.0296 |
| Gray Level Non-Uniformity | GLSZM | HHH | GTV-T | 0.0272 |
| Range | First order | LHH | GTV-N | 0.0269 |
| Large Dependence High Gray Level Emphasis | GLDM | HHL | GTV-N | -0.0224 |
| Contrast | GLCM | LLH | GTV-N | 0.0148 |
| Joint Energy | GLCM | LHH | GTV-N | -0.0128 |
| Cluster Shade | GLCM | LHL | GTV-N | 0.0098 |
| Zone Entropy | GLSZM | LHL | GTV-N | 0.0051 |

**Table E4.** Significant features and their coefficients included in the radiomics signature for perineural invasion prediction

| Feature Name | Feature Class | Wavelet Filter | ROI | Coefficient |
| --- | --- | --- | --- | --- |
| Strength | NGTDM | LHH | GTV-T | 0.7289 |
| High Gray Level Zone Emphasis | GLSZM | LHH | GTV-T | -0.4887 |
| Small Dependence Low Gray Level Emphasis | GLDM | HLH | GTV-T | -0.3625 |
| Small Area Low Gray Level Emphasis | GLSZM | LLL | GTV-N | -0.2767 |
| Maximum | First order | LHH | GTV-T | -0.2479 |
| Inverse Variance | GLCM | LLH | GTV-T | 0.2395 |
| Small Area Low Gray Level Emphasis | GLSZM | HLL | GTV-N | -0.2343 |
| Root Mean Squared | First order | LHL | GTV-N | 0.2083 |
| Short Run Emphasis | GLRLM | - | GTV-T | -0.1231 |
| Dependence Non-Uniformity | GLDM | LHL | GTV-N | -0.1133 |
| Run Length Non-Uniformity Normalized | GLRLM | - | GTV-T | -0.0452 |
| Zone Entropy | GLSZM | HLL | GTV-N | 0.0260 |
| Long Run Low Gray Level Emphasis | GLRLM | LHH | GTV-T | -0.0226 |
| Dependence Entropy | GLDM | HLL | GTV-N | -0.0107 |
